# The Association of Oleic Acid and Dexamethasone Acetate into Nanocapsules Enables a Reduction in the Effective Corticosteroid Dose in a UVB Radiation-Induced Sunburn Model in Mice

**DOI:** 10.3390/pharmaceutics16020176

**Published:** 2024-01-26

**Authors:** Natháli Schopf Pegoraro, Mailine Gehrcke, Camila Camponogara, Maria Fernanda Pessano Fialho, Letícia Cruz, Sara Marchesan Oliveira

**Affiliations:** 1 Graduate Program in Biological Sciences: Toxicological Biochemistry, Centre of Natural and Exact Sciences, Federal University of Santa Maria, Santa Maria 97105-900, RS, Brazil; nathalipegoraro@gmail.com (N.S.P.); milacamponogara@gmail.com (C.C.); mariafpessano@outlook.com (M.F.P.F.); 2 Graduate Program in Pharmaceutical Sciences, Centre of Health Sciences, Federal University of Santa Maria, Santa Maria 97105-900, RS, Brazil; mailine3@hotmail.com (M.G.); leticia.cruz@ufsm.br (L.C.); 3 Department of Biochemistry and Molecular Biology, Centre of Natural and Exact Sciences, Federal University of Santa Maria, Santa Maria 97105-900, RS, Brazil

**Keywords:** skin inflammation, nanocarriers, edema, anti-inflammatory effects

## Abstract

Dexamethasone has a high anti-inflammatory efficacy in treating skin inflammation. However, its use is related to the rebound effect, rosacea, purple, and increased blood glucose levels. Nanotechnology approaches have emerged as strategies for drug delivery due to their advantages in improving therapeutic effects. To reduce dexamethasone-related adverse effects and improve the anti-inflammatory efficacy of treatments, we developed nanocarriers containing this corticosteroid and oleic acid. Nanocapsules and nanoemulsion presented dexamethasone content close to the theoretical value and controlled dexamethasone release in an in vitro assay. Gellan gum-based hydrogels were successfully prepared to employ the nanostructured systems. A permeation study employing porcine skin showed that hydrogels containing non-nanoencapsulated dexamethasone (0.025%) plus oleic acid (3%) or oleic acid (3%) plus dexamethasone (0.025%)-loaded nanocapsules provided a higher amount of dexamethasone in the epidermis compared to non-nanoencapsulated dexamethasone (0.5%). Hydrogels containing oleic acid plus dexamethasone-loaded nanocapsules effectively inhibited mice ear edema (with inhibitions of 89.26 ± 3.77% and 85.11 ± 2.88%, respectively) and inflammatory cell infiltration (with inhibitions of 49.58 ± 4.29% and 27.60 ± 11.70%, respectively). Importantly, the dexamethasone dose employed in hydrogels containing the nanocapsules that effectively inhibited ear edema and cell infiltration was 20-fold lower (0.025%) than that of non-nanoencapsulated dexamethasone (0.5%). Additionally, no adverse effects were observed in preliminary toxicity tests. Our study suggests that nanostructured hydrogel containing a reduced effective dose of dexamethasone could be a promising therapeutic alternative to treat inflammatory disorders with reduced or absent adverse effects. Additionally, testing our formulation in a clinical study on patients with skin inflammatory diseases would be very important to validate our study.

## 1. Introduction

Glucocorticoids started to be used for therapeutic purposes in the mid-20th century; nowadays, they are still widely used worldwide as immunosuppressive and anti-inflammatory agents for their efficacy in treating cutaneous inflammatory disorders [1,2]. However, despite being widely used, glucocorticoids such as dexamethasone (DEX) present several adverse local and systemic effects, including skin atrophy, purpura, ulceration, glaucoma, and increased blood glucose levels with the development of type 2 diabetes mellitus, among others [2,3].

To reduce or avoid drug-related adverse effects, pharmaceutical strategies can be employed, for example, the preparation of nanostructured systems such as nanocapsules and nanoemulsions, employing nanoencapsulation techniques [4]. Nanoencapsulation as a strategy for improving therapy with glucocorticoids has been studied in the last years [5,6,7,8]. Nanocapsules are reservoir systems organized as an oily core surrounded by a polymeric wall, while nanoemulsions are emulsions with droplets in the nanometric range, stabilized by surfactants [9,10]. In addition to reducing adverse effects, nanostructured systems can protect the loaded drug from degradation and prolong the drug release and duration of the pharmacological effects [11,12]. Concerning the benefits of nanocarriers in inflammatory diseases when compared to conventional non-nano drug delivery systems, we also highlight the ability of nanocarriers to allow anti-inflammatory drugs to reach target structures [13].

In fact, previous studies have shown that nanoencapsulation enhances and prolongs the anti-inflammatory effects of different molecules [14,15]. Clinical and preclinical trials have been conducted to reveal the positive impact of these drug carriers. Examples of these trials refer to their anti-inflammatory effects on COVID-19 [16], skin inflammatory disorders [17], osteoarthritis [18], periodontitis [19], and general inflammation-related diseases [20].

Besides the results mentioned above, other alternative biologically active compounds, such as coenzyme Q10 and vitamin E acetate [21], plant derivatives [22,23], and emergent immunotherapies [24,25], effectively reduce skin inflammatory processes. We also previously showed the efficacy of oleic acid (OA), a natural compound present in several vegetable oils [26,27,28,29], in inhibiting skin inflammatory processes induced by UVB radiation and by irritant agent croton oil in mice [30,31]. 

In this study, we hypothesize that a subtherapeutic dose of DEX in association with OA, whose anti-inflammatory efficacy has already been proved, both in nanocarriers, could promote a satisfactory effect in inhibiting skin inflammation, without the occurrence of critical adverse effects related to DEX (MW: 434.5 g mol^−1^, log P: 1.83, insoluble in water at 25 °C). In this sense, this study aimed to develop nanostructure-based hydrogel forms containing the association of oleic acid and DEX into nanocapsules and evaluate their anti-inflammatory effects, employing a UVB-radiation-induced sunburn model in mice.

## 2. Materials and Methods

### 2.1. Materials 

Gellan gum low acyl was kindly donated by CP Kelco (Atlanta, GA, USA). Ethylcellulose 10 (Ethocel™ Standard 10 Premium) was donated by Colorcon^®^ (Cotia, Brazil). Oleic acid (OA) (about 78% purity) was purchased from LabSynth (Diadema, Brazil). Sorbitan monooleate 80 (Span^®^ 80), polysorbate 80 (Tween^®^ 80), hexadecyltrimethylammonium bromide (HTAB), and tetramethylbenzidine (TMB) were obtained from Sigma-Aldrich (São Paulo, Brazil). Dexamethasone acetate was purchased from Nova Derme pharmacy (Santa Maria, Brazil). Ketamine (Dopalen^®^) and xylazine (Anasedan^®^) were obtained from Ceva (Paulínia, Brazil). Sodium citrate, sodium acetate, and acetone were purchased from Vetec (Rio de Janeiro, Brazil). Laboratory kits for biochemical tests were purchased from Labtest Diagnóstica (Lagoa Santa, Brazil), Katal Biotecnologia (Belo Horizonte, Brazil), and Wiener Lab (São Paulo, Brazil). Methanol high-performance liquid chromatography (HPLC) grade was obtained from Merck (Barueri, Brazil). Acetone analytical grade was purchased from Dinâmica Química Contemporânea (Indaiatuba, Brazil). All other reagents and solvents were of analytical grade and used as received.

### 2.2. Methods

#### 2.2.1. Preparation of Nanostructured Dispersions

Nanocapsule and nanoemulsion dispersions were prepared through the interfacial deposition of the preformed polymer [32] and spontaneous emulsification methods, respectively. For the nanocapsules’ preparation, an organic phase containing ethylcellulose 10 (1%), sorbitan monooleate 80 (0.77%), OA (3%), and acetone (27 mL) was prepared and maintained under moderate magnetic stirring at 40 °C for 60 min. After the solubilization of the components, DEX (0.0025 g; 0.025%) was added to the mixture, which was kept under the same conditions for 10 min. Finally, this organic phase was added to 53 mL of an aqueous dispersion of polysorbate 80 (0.77%), and the resultant mixture was kept under moderate magnetic stirring at room temperature for 10 min. The organic solvent and part of the water were removed via evaporation under reduced pressure to obtain a final volume of 10 mL and a DEX concentration of 0.25 mg/mL. For the preparation of the nanoemulsions, the polymeric component ethylcellulose 10 was omitted. For comparative purposes, formulations without DEX were also prepared. The nanodispersions were named as follows: oleic-acid-loaded nanocapsules (NC-B); oleic-acid-loaded nanoemulsion (NE-B); DEX- and oleic-acid-loaded nanocapsules (NC-DEX-OA); and DEX- and oleic-acid-loaded nanoemulsion (NE-DEX-OA).

#### 2.2.2. Physicochemical Characterization of Nanostructured Dispersions

##### pH

To measure the pH values of the nanostructures, the electrode of a calibrated potentiometer (Model pH 21, Hanna Instruments, Barueri, Brazil) was immersed directly in the liquid formulations. The evaluations were performed at room temperature (25 ± 2 °C) and in triplicate (n = 3).

##### Assessment of Particle/Droplet Size, Polydispersity Index, and Zeta Potential of Nanostructured Dispersions 

To evaluate the particle/droplet sizes, polydispersity index (PdI) (n = 3), and zeta potentials (n = 3) of the nanostructured dispersions, the samples were diluted in ultrapure water (1:500) or NaCl (10 mM; 1:500), respectively. The particle/droplet sizes and PdI were assessed using the photon correlation spectroscopy method, while the zeta potentials were determined with microelectrophoresis using ZetaSizer Nano Series (Malvern Instruments, Malvern, UK). 

##### Dexamethasone Acetate Content and Encapsulation Efficiency

The DEX content in the nanocapsules and nanoemulsion dispersions (n = 3) was evaluated by diluting an aliquot of the sample in methanol HPLC grade (10 mL), sonicating over 15 min to extract the drug, and filtrating through a 0.45 μm pore size membrane. Afterwards, the samples were injected into the chromatographic system. The chromatographic method was based on [33]. It consisted of a mobile phase containing a methanol and water mixture (70:30, *v*/*v*) at an isocratic flow rate of 1.0 mL/min, injection volume of 20 μL, DEX detection wavelength of 239 nm, and RP C18 column (Inertsil^®^ ODS-3 5 μm; 4.6 × 150 mm; 100 Å; kept at room temperature) fitted with a guard column (SecurityGuard C18 4.0 × 3.0 mm). The chromatographic system consisted of an LC-10A HPLC system (Shimadzu, Japan) containing an LC-20AT pump, a UV-VIS SPD-M20A detector, a CBM-20A system controller, and a SIL-20A HT valve sample automatic injector. 

For the encapsulation efficiency (EE) assay, the ultrafiltration/centrifugation technique was employed [21]. An aliquot of the dispersions was placed into 10,000 MW centrifugal filter devices (Amicon^®^ Ultra, Millipore, Burlington, MA, USA), and the free drug was separated from the nanostructures via centrifugation at 2200× *g* for 10 min. The resulting ultrafiltrate was diluted and evaluated with the HPLC analysis using the method described above. The EE (%) was calculated as the difference between the total and free concentrations of the DEX determined in the nanodispersions and the ultrafiltrate, respectively, employing the following equation (Equation (1)):

(1) $$\mathrm{EE}=\frac{\mathrm{Total}\;\mathrm{content}-\mathrm{Free}\;\mathrm{content}\;}{\mathrm{Total}\;\mathrm{content}}\;\times \;100$$

#### 2.2.3. In Vitro Drug Release Study

The release profiles of the DEX from the nanocapsules or nanoemulsions were obtained by employing the dialysis bag diffusion technique. An aliquot of 2 mL of the nanostructured samples or methanolic solution of DEX (MS-DEX; 0.25 mg/mL) was placed into 6 cm dialysis bags (molecular weight cut-off 10,000 Da; Sigma-Aldrich, São Paulo, Brazil) and immersed into 100 mL of phosphate buffer at pH 5.5 maintained at 37 °C under uninterrupted moderate magnetic stirring. Aliquots of 1 mL of medium were collected at predetermined times (0.25, 0.5, 0.75, 1, 1.5, 2, 3, 4, 5, 6, 7, 8, 10, 12, and 24 h) for quantification via HPLC of the DEX released. The withdrawn volume was replaced with a fresh medium. 

#### 2.2.4. Preparation of Nanostructure-Based Hydrogels

Hydrogels based on nanostructured systems were prepared using a mortar and pestle by dispersing the polysaccharide gellan gum (2%) in the nanocapsule suspension or nanoemulsion (10 mL). For the hydrogels containing free compounds, DEX and OA were solubilized in dimethyl sulfoxide (DMSO) (close to 3.66%) at the same concentrations found in the nanodispersions and added to an aqueous dispersion of gellan gum (2%). Gellan gum (2%) was dispersed in distilled water to use as the vehicle formulation, using the same methodological process.

#### 2.2.5. Hydrogels Characterization

##### pH

The pH value for the hydrogel aqueous dispersion (10% *w*/*v*) was evaluated using the methodology described in Section pH.

##### Particle/Droplet Size and PdI

The particle sizes and PdI (n = 3) were evaluated using ZetaSizer Nano Series (Malvern Instruments, UK). A hydrogel sample (0.1 g) was diluted in 50 mL of ultrapure water and stirred for 40 min before analysis.

##### Dexamethasone Acetate Content

The DEX content in the hydrogels was evaluated employing the chromatographic method described in Section Dexamethasone Acetate Content and Encapsulation Efficiency. For sample preparation, 0.5 g of hydrogel was solubilized into 25 mL of methanol, submitted to sonication for 30 min, and filtered through a 0.45 μm membrane before analysis.

##### Determination of Spreadability

The parallel plate method was used to evaluate the spreadability of the hydrogels [34,35]. An aliquot of the sample was put in the central hole of a mold glass plate, which was positioned on the surface of a scanner (HP Deskjet, model 3050, J610 series). Ten glass plates with known weights were placed upon the sample after mold glass remotion. Each glass plate was added with an interval of 1 min of the subsequent plate. Using a desktop scanner, at each 1 min interval, hydrogel images were captured, while Image J software (Version 1.45e, National Institutes of Health, Bethesda, MD, USA) captured the spreading areas of the hydrogel. For all formulations, the spreadability factor (SF), the formulation’s ability to expand on a smooth horizontal surface when a gram of weight is added, was calculated. The spreadability factor was calculated by the below equation (Equation (2)):

(2) $$\mathrm{SF}=\frac{A}{W}$$

SF means the spreadability factor (mm^2^/g), A means the maximum spread area (mm^2^) after the addition of the total number of plates, and W is the total weight added (g) on the hydrogel. 

##### Evaluation of Bioadhesive Properties

The bioadhesive properties of the hydrogels were determined by employing porcine ear skin tissue as the biological membrane, which was obtained through donation from a local slaughterhouse (Santa Maria, Brazil). Skin samples were cleaned to remove hair and adipose tissue and stored frozen until use. On the day of the experiment, the skin was acclimated to room temperature for 30 min before the assay. The porcine skin tissue (n = 3) was fixed on the apparatus surface. A traditional balance was used, where one arm contained the probe, added an amount of 0.8 g of sample, while the other arm contained a plastic bottle. The probe was carefully placed in contact with the skin. Water was added to the bottle after one minute. The bioadhesion for each hydrogel was determined by the water volume necessary to detach the probe from the skin surface. A Carbopol^®^ Ultrez hydrogel (0.5%) (G-CBP) was employed with a comparative purpose. The results were expressed as Dynes/cm^2^ considering the skin surface area [36].

#### 2.2.6. Skin Permeation Study

The DEX skin permeation from the hydrogels was assessed by employing the porcine skin tissue obtained from a local slaughterhouse (Santa Maria, Brazil). The skin was cleaned by removing bristles and adipose tissue and stored frozen until its use. Franz diffusion cells were employed for this evaluation. The skin sample was positioned in the Franz cells (n = 6) with the dermis in contact with the receptor medium (phosphate buffer at pH 5.5 maintained at 32 ± 0.5 °C under constant magnetic stirring), while the tested hydrogels (0.5 g) were deposited on the stratum corneum. Twelve hours after incubation, the skin samples and the remaining hydrogel above them were carefully removed, and the receptor medium was collected. Eighteen plastic tapes (Flax^®^, Brazil) were used for the tape stripping technique to remove the stratum corneum. The skin samples were weighed before and after each separation step. The plastic tapes were immersed in 24 mL of methanol, vortexed for 2 min, and submitted to sonication (15 min) and centrifugation (3000 rpm for 15 min). After, the samples were filtered and analyzed using chromatography. For dermis and epidermis separation, the skin samples were immersed in a water bath at 60 °C for 45 s; the epidermis was removed with a spatula [37,38] and inserted into 1 mL of methanol. The remaining dermis was cut out and immersed in 2 mL of methanol. The dermis and epidermis samples were also submitted to vortexing (2 min), sonication (15 min), and centrifugation (3000 rpm for 15 min), filtered and analyzed using chromatography.

#### 2.2.7. In Vivo Evaluation

##### Animals

The Central Animal Facility from the Federal University of Santa Maria produced and supplied male Swiss mice (25–30 g) for all in vivo experimental protocols. The mice were kept under a controlled temperature (22 ± 2 °C) and on a 12 h light–dark cycle, and received standard laboratory chow and water ad libitum. Before the behavioral tests, the mice were acclimatized to the experimental room for at least one hour, and the experimental procedures were performed between 8:00 a.m. and 5:00 p.m. All experimental protocols followed the national legislation (Guidelines of Brazilian Council of Animal Experimentation—CONCEA) and Animal Research: Reporting In Vivo Experiments ARRIVE guidelines [39]. The experimental procedures were approved by the Institutional Animal Care and Use Committee of the Federal University of Santa Maria (4369251019/2019). 

The mice were randomly divided into the following experimental groups containing 6 animals each: naïve (non-irradiated); UVB radiation (UVB 0.5 J/cm^2^); UVB 0.5 J/cm^2^ + G-Vehicle (vehicle hydrogel); UVB 0.5 J/cm^2^ + G-L-DEX-OA (hydrogel containing non-nanoencapsulated DEX at 0.025% and non-nanoencapsulated oleic acid at 3%); UVB 0.5 J/cm^2^ + G-NC-B (hydrogel containing oleic-acid-loaded nanocapsules); UVB 0.5 J/cm^2^ + G-NC-DEX-OA (hydrogel containing oleic-acid- and DEX-loaded nanocapsules); and UVB 0.5 J/cm^2^ + G-L-DEX (hydrogel containing non-nanoencapsulated DEX at 0.5%; positive control).

##### Antiedematogenic Efficacy

UVB radiation was employed to induce the acute ear edema model in the mice, according to [30]. Briefly, the mice were anesthetized with 90 mg/kg of ketamine + 3 mg/kg of xylazine through a single intraperitoneal injection [40], and subsequently, they were allocated to a bench under the lamp. The mice’s right ears were exposed to UVB radiation for 30.8 min. The remaining body surface was protected from UV radiation with a cotton cloth. The UVB irradiation rate and the dose employed were 0.27 mW/cm^2^ and 0.5 J/cm^2^, respectively [21]. 

The surface of the right mouse ear was topically treated with hydrogels (15 mg/ear) immediately after the UVB irradiation, according to the groups of treatment described above [30]. 

A digital micrometer (Digimess, São Paulo, Brazil) was used to measure the mouse right ear thickness. The measurements were performed before (basal measure) and 24 h after exposure to UVB radiation or UVB radiation plus topical treatments. The micrometer was positioned near the tip of the ear, distal to the cartilaginous ridges. When compared to the basal value, an increase in ear thickness after UVB radiation was considered to be edema, expressed in μm as the difference between the basal thickness and ear thickness at 24 h after UVB or UVB plus treatments. A single experimenter performed this analysis to reduce experimental bias [21,30].

##### Assessment of Myeloperoxidase (MPO) Activity

The evaluation of inflammatory cell infiltration was performed using the MPO activity determination. The mice were euthanized 24 h after UVB radiation or UVB radiation plus treatments to collect their ear samples. A solution containing acetate buffer (80 mM, pH 5.4) and 0.5% hexadecyltrimethylammonium bromide (HTAB) was used to homogenize the ear samples. After homogenization, the samples were immediately centrifuged at 16.000× *g* at 4 °C for 30 min. The supernatants obtained were incubated at 37 °C for 10 min with a solution containing acetate buffer and tetramethylbenzidine (TMB) solution (18.4 nM) [41]. The absorbance of the samples was obtained spectrophotometrically at 630 nm, and the results were expressed as the optical density (OD)/mL of the sample.

##### Evaluation of Hydrogels’ Antiedematogenic Efficacy over 72 h

We also verified the ability of the nanostructured hydrogels to sustain their antiedematogenic effect for 72 h. For this, the mouse right ear was exposed to UVB radiation and immediately treated with a single application of the hydrogels, as described above. The ear thickness was measured before (basal measure) and at 24 h, 48 h, and 72 h after the UVB radiation and topical treatments, as described in Section Antiedematogenic Efficacy.

##### Histological Analysis

A histological analysis of the ear tissue was performed 72 h after UVB radiation to investigate the inflammatory cell infiltration to the ear tissue irradiated or irradiated and treated with hydrogels. The mice were euthanized after the ear edema measurement assessment, and the right ear was collected and fixed in Alfac solution (16:2:1 mixture of ethanol 80%, formaldehyde 40%, and acetic acid). To section the ear samples at 5 μm and stain them with hematoxylin-eosin, they were embedded in paraffin. A quantitative analysis of the inflammatory cell number was performed in a representative area selected using 10 × objectives [30]. This analysis was performed blindly. The counting of the inflammatory cells per field was performed using the Image J software. Three fields from six distinct histological slides of each group were analyzed.

##### Biochemical Markers of Toxicity

As indicators of hepatic and pancreatic alterations, we evaluated the alanine transaminase (ALT) and aspartate transaminase (AST) activities and blood glucose levels, respectively. Seventy-two hours after inducing and treating the inflammatory process, the mice were euthanized and blood samples were collected via cardiac puncture. To obtain the serum, the blood samples were centrifuged at 3000 rpm for 10 min. Labtest^®^, Katal^®^, and Wiener^®^ Lab kits were used to assess the AST and ALT activities and the glucose levels using spectrophotometry, according to the manufacturer’s specifications (Labtest Diagnostica, Lagoa Santa, Brazil; Katal Biotecnologica, Belo Horizonte, Brazil; Wiener Laboratórios, São Paulo, Brazil) [42].

#### 2.2.8. Statistical Analysis

All statistical tests were conducted using the GraphPad Prism 6.00 or GraphPad Prism 8.00 Software (San Diego, CA, USA). The results are presented as the mean + standard error of the mean (SEM) or as the mean ± standard deviation (SD) for the formulation development assays. The results are also reported as geometric means plus their respective 95% confidence limits. The inhibitory effect was calculated based on the control groups, whose results were considered 100% of the effect. One-way or two-way (repeated measures) analyses of variance (ANOVA) followed by Tukey’s post hoc test were used to evaluate the statistical significance between groups. P-values less than 0.05 (*p* < 0.05) were considered as significative. 

## 3. Results

### 3.1. Characterization of Nanostructured Dispersions

**Figure 1:**
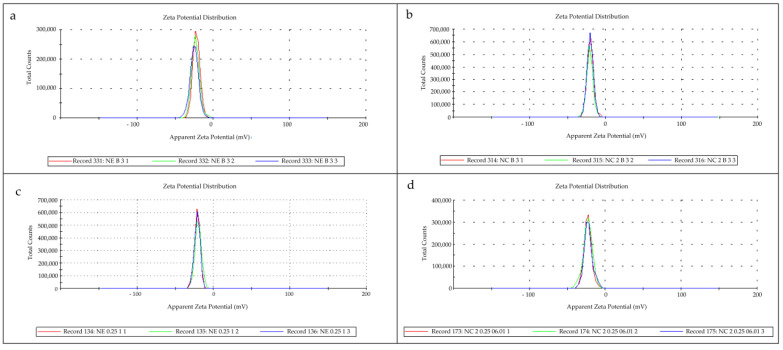
Representative images of the zeta potential determined via microelectrophoresis of the nanocarriers NE-B (**a**), NC-B (**b**), NE-DEX-OA (**c**), and NC-DEX-OA (**d**).

Table 1 presents the results of the nanostructured dispersions’ characterization. The pH values obtained were in the acidic range for all dispersions. The nanostructures obtained were in the nanometric range (close to 200 nm) with a low PdI value (lower than 0.2), indicating a narrow size distribution. The zeta potential was in the negative range, close to −20 mV for all formulations (Figure 1). The DX content for all the nanostructured systems was close to the theoretical value (0.25 mg/mL). The DEX encapsulation efficiency was 95.47% for NC-DEX-OA and 94.58% for NE-DEX-OA. 

### 3.2. In Vitro DEX Release from Nanostructures

**Figure 2:**
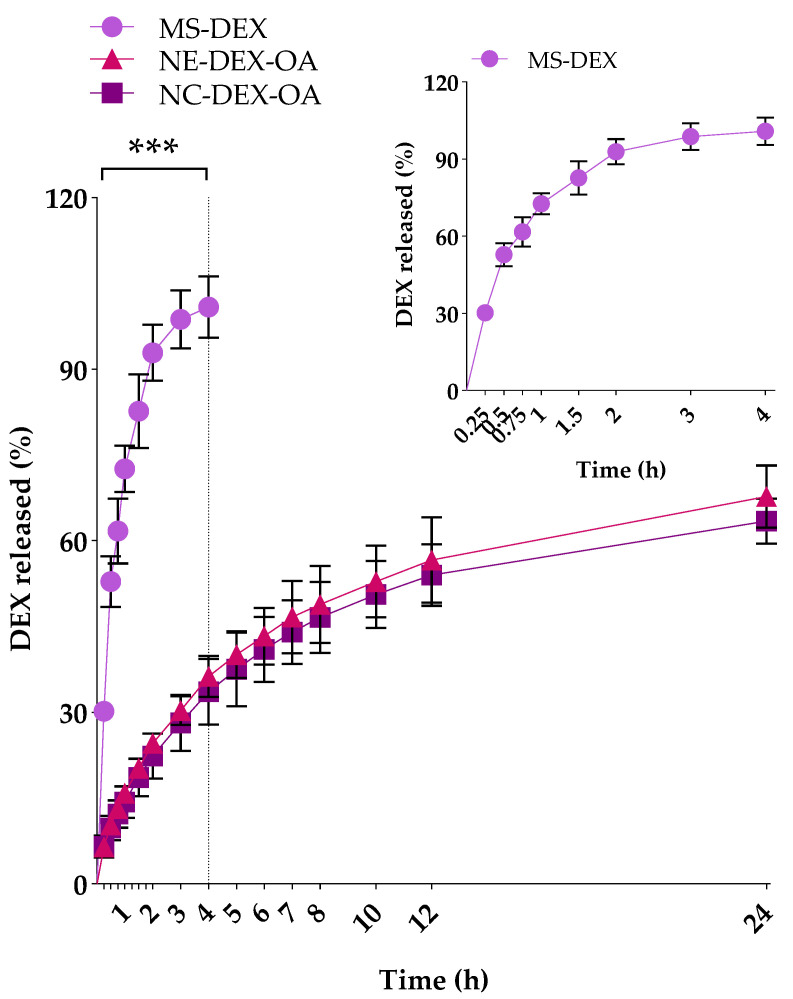
In vitro DEX release profiles from nanocapsules (NC-DEX-OA), nanoemulsion (NE-DEX-OA), and methanolic solution (MS-DEX) between 0 and 24 h incubation. Data represent the mean ± SD (n = 3) and were analyzed with two-way ANOVA followed by Tukey’s post hoc test. *** *p* < 0.001 indicates a significant difference compared to NC-DEX-OA and NE-DEX-OA.

The release profiles of DEX from the nanocapsules, nanoemulsion, and methanolic solution are represented in Figure 2. The release profiles were obtained by collecting and analyzing samples between 0 and 24 h of incubation. We observed that all the DEX content was released from MS-DEX until the 4 h assay (100.87 ± 5.40%), evidenced by the dotted in the Figure 2. On the other hand, the nanostructures exhibited an ability to control the DEX release: 63.46 ± 3.98% of DEX was released from NC-DEX-OA after the 24 h assay, while 67.75 ± 5.44% of DEX was released from NE-DEX-OA at the same time point. 

### 3.3. Hydrogels Characterization

Table 2 presents the characterization of the hydrogels developed based on the nanodispersions. After the dispersion of gellan gum into the nanostructured systems, the pH values were maintained in the initial range, close to 5.0. The mean diameter of the nanocarriers after the redispersion of the hydrogel in water was close to the initial value. The mean diameter for the nanocapsules-based hydrogel G-NC-DEX-OA was lower than the respective nanoemulsion-based hydrogel G-NE-DEX-OA (*p* < 0.01). The aqueous dispersion of gellan gum (G-Vehicle) resulted in colloidal structures with a higher mean diameter and size distribution when compared to the other hydrogels (*p* < 0.001). The DEX content was close to 100% for all hydrogels.

### 3.4. Evaluation of Hydrogels Bioadhesive Properties

**Figure 3:**
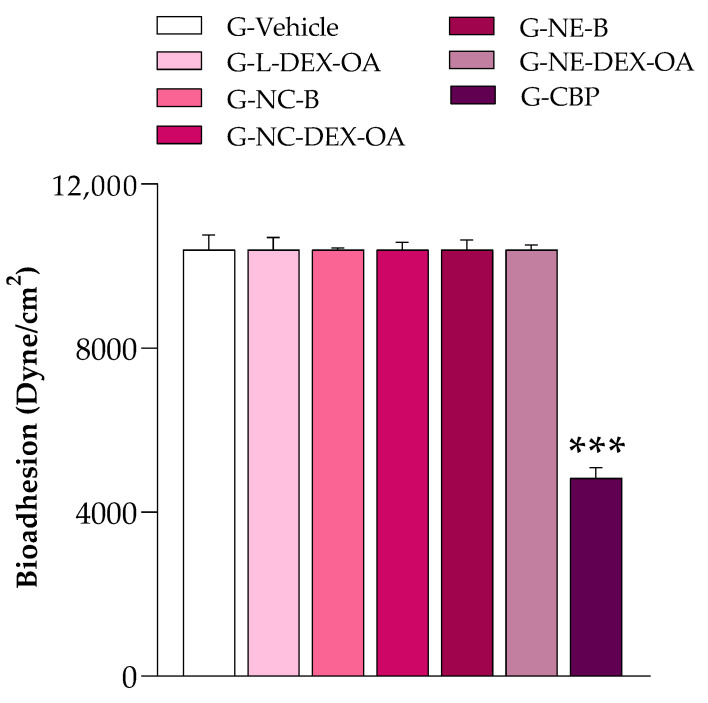
Bioadhesive study for the gellan-gum- and Carbopol^®^ Ultrez (CBP)-based hydrogels. Data represent the mean ± SD (n = 3) and were analyzed with one-way ANOVA followed by Tukey’s post-hoc test. *** *p* < 0.001 indicates a significant difference from all other hydrogels tested.

The results of the bioadhesive study are presented in Figure 3. We observed that the necessary force to detach all the developed gellan-gum-based hydrogels of the skin was significantly higher (*p* < 0.001) than that necessary to detach the Carbopol^®^ Ultrez hydrogel of the porcine skin membrane. 

### 3.5. DEX Skin Permeation Study

**Figure 4:**
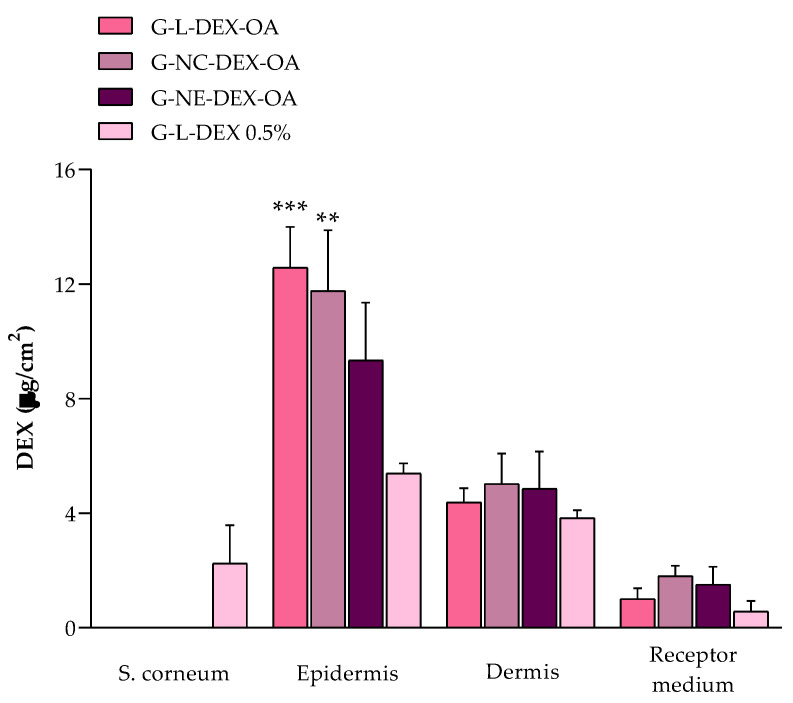
In vitro permeation study of DEX from hydrogels in porcine skin tissue. Distribution of DEX through the skin layers 12 h after incubation with hydrogels. Results are expressed as mean + SD from 3 independent experiments (n = 6). Data were analyzed with one-way ANOVA followed by Tukey’s post hoc test. ** *p* < 0.01 and *** *p* < 0.001 indicate significant differences of G-L-DEX-OA and G-NC-DEX-OA, respectively, compared to G-L-DEX 0.5% in the epidermis layer.

The skin permeation of the DEX from the hydrogels was also assessed. Figure 4 represents the DEX distribution through the porcine skin layers. All the tested hydrogels provided a higher DEX amount in the epidermis than in the other skin layers. Moreover, the DEX from G-L-DEX-OA, G-NC-DEX-OA, and G-NE-DEX-OA was not quantified in the stratum corneum. The total DEX amounts that permeated through the skin layers were 53.26 ± 12.02 μg/g, 52.72 ± 19.54 μg/g, 44.57 ± 23.76 μg/g, and 36.03 ± 8.37 μg/g for G-L-DEX-OA, G-NC-DEX-OA G-NE-DEX-OA, and G-L-DEX, respectively.

### 3.6. In Vivo Anti-Inflammatory Efficacy

#### 3.6.1. Assessment of Antiedematogenic Effect and Enzyme MPO Activity

**Figure 5:**
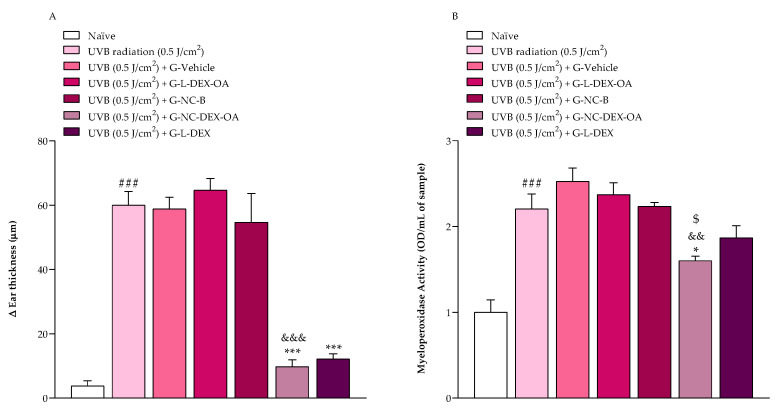
(**A**) Antiedematogenic effect of topically applied (15 mg/ear) hydrogels containing OA and DEX on acute UVB-radiation-induced sunburn model in mice. (**B**) Effect of hydrogels containing oleic acid and dexamethasone acetate on the MPO enzyme activity in the mice ear tissue after acute UVB-radiation-induced sunburn model. All formulations were applied in mice’s right ear immediately after UVB radiation. Ear thickness and MPO activity were measured 24 h after UVB radiation or UVB radiation plus treatments. Each bar represents the mean + SEM (n = 6); ^###^ *p* < 0.001 shows a significant difference compared to the naïve group; *** *p* < 0.001 shows a significant difference compared to the UVB radiation group; ^&&&^ *p* < 0.001 shows a significant difference compared to the G-L-DEX-OA and G-NC-B groups; ^&&^ *p* < 0.01 indicates a significant difference compared to the G-L-DEX-OA group; and ^$^ *p* < 0.05 refers to the significant difference when compared to the G-NC-B group. One-way ANOVA followed by post hoc Tukey’s test.

Exposure to UVB radiation increased the mice’s ear thickness by 60 ± 4 μm, characterized as ear edema. G-NC-DEX-OA and G-L-DEX reduced the ear edema, with inhibitions of 89.26 ± 3.77% and 85.11 ± 2.88%, respectively. None of the other tested hydrogels reduced the UVB-radiation-induced ear edema (Figure 5A). 

In addition to the antiedematogenic activity, we investigated if the hydrogels could inhibit the inflammatory cell infiltration to damaged tissue by measuring the MPO enzyme activity. UVB radiation increased the MPO activity in the mice ear tissue compared to the naïve group (non-irradiated). Topical G-NC-DEX-OA and G-L-DEX inhibited the MPO activity on the mice’s ear tissue by 49.58 ± 4.29% and 27.60 ± 11.70%, respectively (Figure 5B).

#### 3.6.2. Evaluation of Hydrogels’ Antiedematogenic Efficacy through 72 h

**Figure 6:**
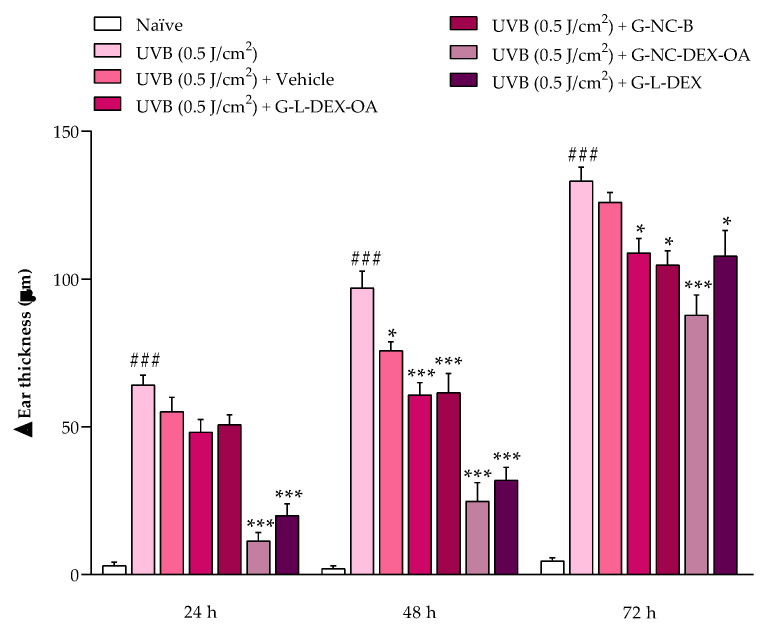
Antiedematogenic effect of the hydrogels containing OA and DEX on the UVB radiation-induced ear edema in mice. All formulations (15 mg/ear) were applied immediately after UVB radiation. Ear thickness was measured at 24 h, 48 h, and 72 h after ear UVB radiation or UVB radiation plus treatments with the hydrogels. Each bar represents the mean + SEM (n = 5–6); ^###^ *p* < 0.001 shows a significant difference compared to the naïve group; * *p* < 0.05 and *** *p* < 0.001 show a significant difference compared to the UVB (0.5 J/cm^2^) (no treatment) group. One-way ANOVA followed by post hoc Tukey’s test.

UVB radiation increased the mice’ ear thickness by 64 ± 3 µm, 97 ± 6 µm, and 133 ± 5 µm at 24 h, 48 h, and 72 h after UVB exposure, respectively. Twenty-four hours after UVB radiation or UVB radiation plus treatments, G-NC-DEX-OA reduced the mice ear edema with an 86.27 ± 4.58% inhibition. At the same time, the positive control, G-L-DEX, inhibited it by 72.22 ± 6.43%, respectively, compared to the irradiated group (no treatment). Forty-eight hours after UVB radiation or UVB radiation plus treatments, the ear edema was reduced by G-Vehicle, G-L-DEX-OA, and G-NC-B, with inhibitions of 22.28 ± 3.08%, 38.11 ± 4.38%, and 37.26 ± 6.78%, respectively. Importantly, at this time, G-L-DEX reduced ear edema by 68.42 ± 4.49%, while the treatment with G-NC-DEX-OA presented an inhibition of 76.00 ± 6.66%. At 72 h after UVB radiation or UVB radiation plus treatments, G-L-DEX-OA, G-NC-B, and G-L-DEX were capable of reducing ear edema by 18.99 ± 3.81%, 22.10 ± 3.68%, and 19.76 ± 6.72%, respectively, while G-NC-DEX-OA presented a better antiedematogenic effect with 35.32 ± 5.30% inhibition (Figure 6).

#### 3.6.3. UVB-Radiation-Induced Inflammatory Cell Infiltration 

**Figure 7:**
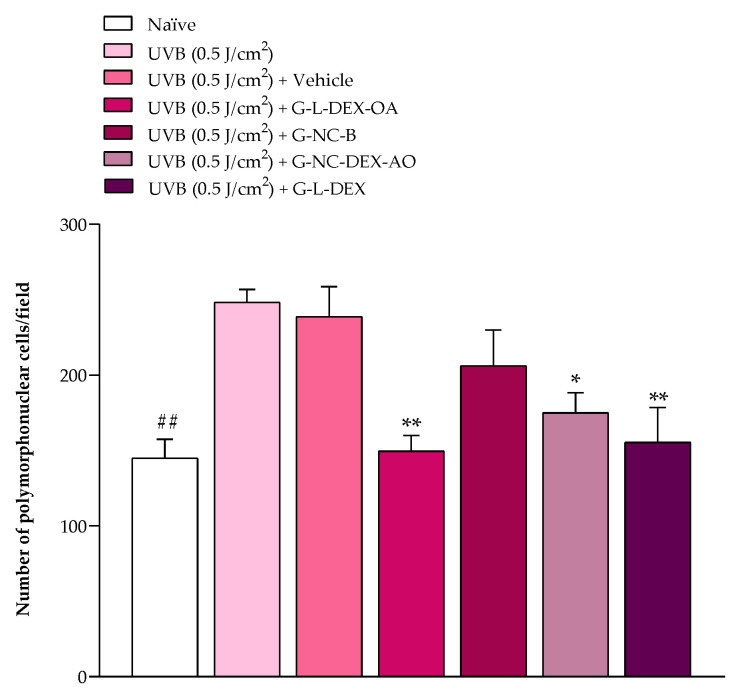
Effect of hydrogels containing OA and DEX on the inflammatory cell infiltration in mice’s right ear tissue employing histological technique. Polymorphonuclear cells per field of the mice’s right ear tissue were quantified at 72 h after the UVB radiation or UVB radiation plus treatments. Each bar represents the mean + SEM (n = 5–6). ^##^ *p* < 0.01 indicates a significant difference compared to the naïve group; * *p* < 0.05 and ** *p* < 0.01 denote a significant difference compared to the UVB radiation group. One-way ANOVA followed by post hoc Tukey’s test.

The histological analysis of the mice’s ear tissue at 72 h after UVB radiation or UVB radiation plus treatments revealed that the UVB radiation increased cell infiltration to the ear tissue (248 ± 8 inflammatory cells per field) when compared to the naïve group (145 ± 12 inflammatory cells per field) (Figure 7). Topical treatment with G-NC-DEX-OA and G-L-DEX-OA inhibited the cell infiltration by 70.87 ± 13.02% and 95.47 ± 9.84%, respectively; the positive control G-L-DEX inhibited this inflammatory parameter by 89.90 ± 22.36%. 

#### 3.6.4. Preliminary Adverse Effects Assessment 

Three biochemical markers were also evaluated as signs of toxicity: AST and ALT enzyme activities and glucose levels. No statistical difference was observed in the biochemical marker levels on the blood samples of animals treated with the G-NC-B, G-NC-DEX-OA, or G-L-DEX (Table 3). 

## 4. Discussion

Many natural oils are topically used worldwide due to the facility of obtention. These natural oils present biological activities like antimicrobial, antioxidant, anti-inflammatory, and others [43]. OA is a natural compound found in several oils (olive oil, grape seed oil, and avocado oil) [26,27,28,29,44] and has shown beneficial properties for skin health and disease [43,45,46]. Our research group have already demonstrated the OA potential to inhibit skin inflammatory processes in UVB-radiation-induced and irritant agent croton oil inflammation models, both in mice [30,31].

Skin inflammatory processes are complex, involve many molecular and cellular mediators, and present the risk of becoming chronic. For these reasons, inflammatory processes must be finely controlled, which is quite difficult in clinical practice [47,48]. DEX and other topical corticosteroids are broadly employed to contain inflammatory diseases [48], although using these drugs causes severe adverse effects [3].

We previously observed that repeated topical treatment with a hydrogel containing 0.5% DEX for nine consecutive days in the mouse’s right ear induced a significant increase in glucose blood levels [31], a classical adverse effect related to the use of corticosteroids [3]. In the present study, we evaluated if a subtherapeutic dose of DEX, associated with OA, could reduce skin inflammation without causing adverse effects. Additionally, we combined nanostructured systems to vehicle both these compounds.

In the first moment, we prepared and characterized nanoemulsions and nanocapsule suspensions containing DEX and OA. Additionally, we tested the in vitro DEX release from these systems to simulate DEX release in vivo. Nanoemulsions and nanocapsules presented the ability to control the DEX release over 24 h. Our result is relevant since developing a system that can control drug release by employing nanotechnology is extremely positive once it improves the treatment schedule by enabling an increase in the administration interval. This fact contributes to reducing the occurrence of adverse effects [49], which is particularly interesting about drugs with severe adverse effects like DEX. Moreover, delivering drugs in a spatiotemporally controlled manner can potentially improve patient adherence to therapeutic regimen [50,51].

Subsequently, we prepared gellan-gum-based hydrogels containing the nanostructured systems with DEX and OA. Hydrogels are pharmaceutical dosage forms for drug delivery, bringing benefits such as ease of application and capability to vehicle a wide variety of drugs, besides avoiding the first-pass effect in the organism [52,53].

Important parameters must be considered in the semisolid’s development: spreadability, bioadhesion, and drug permeation through skin layers. We obtained spreadability values close to 0.5 for all hydrogels developed; that is, the lower the spreadability value, the lower the formulation ability to spread over the applied area, and the higher the retention at the local of the lesion [54]. In fact, such behavior causes a gradual and slow release of the active from the formulation to the skin layers. Additionally, the higher retention at the local of the lesion is an important feature of pharmaceutical formulations intended for skin inflammatory disease treatment [55,56]. Similar results for this parameter were obtained in other studies that showed important anti-inflammatory effects, employing semisolids of different nanostructured systems and loaded drugs [14,21]. Further, all hydrogels presented higher bioadhesive behavior compared to the Carbopol^®^ Ultrez hydrogel, which is a recognized gel-forming polymer for providing mucoadhesion to pharmaceutical formulations [57,58]. These results suggest the ability to be in close contact with the absorption tissue for longer when applied and promote local drug release [59,60]. Improving the time of contact between the drug delivery system and the wound can contribute to wound moisture maintenance, a recognized important factor of ideal wound care therapies [61,62]. Additionally, mucoadhesive drug delivery systems can increase drug bioavailability, favoring wound healing [59] Furthermore, a bioadhesive delivery system with controlled drug release could help to maintain an effective concentration of the drug at the action site [57,59,63]. A similar result was already evidenced in a previous study of our research group [36]. Through the permeation study, we observed that the DEX released from the hydrogels was retained, for the most part, in the epidermis layer. At the same time, an intermediate amount reached the dermis layer, and consequently, a lower amount was available to suffer systemic absorption. A higher amount of drug retained in the epidermis layer can be an important approach to wound treatment since burns affect the epidermis layer first [64]. A higher amount of drug retained in the epidermis can prevent the burn wound from advancing into a deeper layer, becoming a deeper burn [64]. Since substantial DEX-related adverse effects occur after its systemic absorption, avoiding this event is extremely important [65].

No difference was observed between the nanocapsules and nanoemulsions in relation to the DEX in the in vitro release test, bioadhesion, and DEX skin permeation. However, it is already known that polymeric nanoparticles for drug delivery present some advantages over nanoemulsions, since the polymer can directly influence the nanosuspension and loaded drug stability. Importantly, nanocapsules can also contribute to a drug-controlled release [10]. In this context, we chose the nanocapsules formulation containing the association of AO and DEX for the in vivo anti-inflammatory activity evaluation in a UVB-radiation-induced skin inflammation model.

UVB radiation exposure triggers skin inflammation that involves processes such as vasodilation, which leads to edema development, erythema, inflammatory cell infiltration, inflammatory cytokines/chemokines release, epidermal hyperplasia, and oxidative stress, besides skin photoaging and photocarcinogenesis [15,21,22,30,66,67]. So, restricting acute skin inflammation is extremely important to avoid it from becoming chronic.

Topical G-NC-DEX-OA presented a high anti-inflammatory efficacy by reducing mice’s ear edema and leukocyte infiltration induced by UVB radiation. These results are important, since edema formation is often the first and essential marker of skin inflammation [68,69]. Furthermore, inflammatory skin diseases are characterized by intense leukocyte infiltration, a defense mechanism in inflammatory processes [70]. Thus, since G-NC-DEX-OA reduced the ear edema and leukocyte infiltration caused by UVB radiation, our results indicate a possible application of G-NC-DEX-OA to treat human inflammatory diseases. Additionally, a single G-NC-DEX-OA application sustained this anti-inflammatory effect for at least 72 h after exposure to UVB radiation. As mentioned above, sustaining the drug release and effect enables an increase in the administration interval, favoring the patient adhesion to the pharmacological treatment. Our results are according to previous studies, which demonstrated antiedematogenic and anti-inflammatory effects of topical OA and DEX after mouse ear exposure to UVB radiation [22,30]. Relevantly, we demonstrated that hydrogels containing OA- plus DEX-loaded nanocapsules in a subtherapeutic dose presented sustained antiedematogenic and anti-inflammatory effects, without causing adverse effects in the preliminary toxicity tests. Given the complexity of inflammatory processes, combining both these actives made it possible to reduce DEX to a subtherapeutic dose, which is a promising approach to improving anti-inflammatory effects while diminishing the possibility of dose-related adverse effects.

The activity of the AST and ALT enzymes in the blood serum are commonly used as markers of liver damage, and the long-term application of dexamethasone and other glucocorticoids is known to upregulate the activity of these enzymes [71,72]. In this study, neither the single topical application of the hydrogel containing non-nanoencapsulated DEX nor the hydrogel containing oleic-acid- and DEX-loaded nanocapsules increased the activity of the AST and ALT enzymes. Additionally, repeated use of glucocorticoids such as dexamethasone is also related to increased blood glucose levels [3,29]. However, a single topical application of the treatments did not increase the mice’s blood glucose levels. Although non-nanoencapsulated DEX raised blood glucose levels when compared to the other treatments, this increase was not significant. The discrepancies between our results and the results found in previous studies probably occurred due to the time of the administration of the treatments, where we performed a single topical application of the formulations, while previous studies have demonstrated adverse effects of dexamethasone after long-term treatment. In this sense, more studies considering the toxicity of the formulations tested here are necessary to guarantee the absence of adverse effects when applied in the long term.

## 5. Conclusions

We successfully developed nanocapsule suspensions and nanoemulsions containing OA and DEX, since they could control DEX release in an in vitro assay. A DEX permeation study employing porcine skin showed that hydrogels provided more DEX in the epidermis, which can guarantee the anti-inflammatory effect of the formulation without it reaching the bloodstream and causing systemic adverse effects. G-NC-DEX-OA effectively reduced the inflammatory damage after mice skin exposure to UVB radiation with an employed DEX dose 20-fold lower than non-nanoencapsulated (positive control), another fact that could reduce the probability of DEX to cause adverse effects, since the dose was significantly reduced. Since this model mimics solar sunburn, hydrogels containing OA- plus DEX-loaded nanocapsules could represent a prosperous option for treating burns, especially sunburns. Thus, testing our formulation in a clinical study on patients with skin inflammatory diseases would be essential to validate our study.

## Figures and Tables

**Table 1:** pH, mean diameter, polydispersity index, zeta potential, and DEX content for the nanodispersions.

Formulation	pH	Mean Diameter (nm)	PdI	Zeta Potential (mV)	DEX Content (%)
NC-B	5.0 ± 0.1	176 ± 10	0.09 ± 0.06	−20.0 ± 0.5	-
NC-DEX-OA	5.2 ± 0.1	175 ± 14	0.09 ± 0.03	−19.8 ± 3.9	100.29 ± 2.44
NE-B	4.6 ± 0.2	208 ± 19	0.14 ± 0.06	−20.8 ± 2.2	-
NE-DEX-OA	4.7 ± 0.3	226 ± 46	0.19 ± 0.08	−22.2 ± 3.4	98.59 ± 3.95

Each value represents the mean ± SD (n = 3). Data were analyzed with one-way ANOVA followed by Tukey’s post hoc test.

**Table 2:** pH, mean diameter, polydispersity index, DEX content, and spreadability factor for the hydrogels obtained from the nanodispersions.

Formulation	pH	Mean Diameter (nm)	PdI	DEX Content (%)	SF (mm^2^/g)
G-Vehicle	5.0 ± 0.2	1345 ± 436 ***	0.93 ± 0.06 ***	-	0.68 ± 0.08
G-L-DEX-OA	5.2 ± 0.1	-	-	100.61 ± 3.19	0.48 ± 0.09
G-NC-B	5.4 ± 0.2	195 ± 06	0.20 ± 0.01	-	0.59 ± 0.05
G-NC-DEX-OA	5.4 ± 0.1	181 ± 20	0.17 ± 0.08	97.23 ± 2.73	0.46 ± 0.05 ^&^
G-NE-B	5.1 ± 0.1	218 ± 28	0.24 ± 0.03	-	0.57 ± 0.09
G-NE-DEX-OA	5.3 ± 0.1	280 ± 38	0.29 ± 0.10	96.72 ± 1.57	0.51 ± 0.07

Each value represents the mean ± SD (n = 3). *** *p* < 0.001 denotes the significant difference of G-Vehicle compared with the other formulations; ^&^ *p* < 0.05 indicates a significant difference when compared to G-Vehicle. One-way ANOVA followed by post hoc Tukey’s test.

**Table 3:** Biochemical markers levels on blood samples of animals treated with hydrogels.

Formulation	AST (U/L)	ALT (U/L)	Glucose (mg/dL)
G-NC-B	63 ± 37	31 ± 18	163 ± 94
G-NC-DEX-OA	57 ± 25	28 ± 12	145 ± 65
G-L-DEX	59 ± 26	34 ± 15	183 ± 82

Each value represents the mean ± SEM (n = 5–6). One-way ANOVA followed by post hoc Tukey’s test.

## Data Availability

The data can be shared up on request.

## Abbreviations

ANOVA—analysis of variance; CONCEA—Brazilian Council of Animal Experimentation; DEX—dexamethasone acetate; DMSO—dimethyl sulfoxide; EE—encapsulation efficiency; G-CBP—Carbopol Ultrez^®^ 0.5% hydrogel; G-L-DEX—hydrogel containing non-nanoencapsulated dexamethasone acetate at 0.5%; G-L-DEX-OA—hydrogel containing non-nanoencapsulated dexamethasone acetate at 0.025% and non-nanoencapsulated oleic acid at 3.0%; G-NC-B—hydrogel containing oleic acid-loaded nanocapsules; G-NC-DEX-OA—hydrogel containing oleic acid and dexamethasone acetate-loaded nanocapsules; G-Vehicle—vehicle hydrogel (gellan gum aqueous dispersion); HPLC—high performance liquid chromatography; HTAB—hexadecyltrimethylammonium bromide; Imax—maximum inhibition; MPO—myeloperoxidase; MS-DEX—dexamethasone acetate methanolic solution; NC-B—nanocapsules containing free (non-nanoencapsulated) oleic acid; NC-DEX-OA—dexamethasone acetate and oleic acid-loaded nanocapsules; NE-B—nanoemulsion containing free (non-nanoencapsulated) oleic acid; NE-DEX-OA—dexamethasone acetate and oleic acid-loaded nanoemulsion; OA—oleic acid; OD—optical density; PdI—polydispersity index; SD—standard deviation; SEM—standard error of the mean; SF—spreadability factor; TMB—tetramethylbenzidine; UVB—ultraviolet radiation type B.

## References

[1] Fathallah N., Slim R., Larif S., Hmouda H., Ben Salem C. (2015). Drug-Induced Hyperglycaemia and Diabetes. Drug Saf..

[2] Johns E.C., Reynolds R.M. (2019). Topical Glucocorticoids and Risk of Type 2 Diabetes Mellitus. Nat. Rev. Endocrinol..

[3] Coondoo A., Phiske M., Verma S., Lahiri K. (2014). Side-Effects of Topical Steroids: A Long Overdue Revisit. Indian Dermatol. Online J..

[4] Frank L.A., Contri R.V., Beck R.C.R., Pohlmann A.R., Guterres S.S. (2015). Improving Drug Biological Effects by Encapsulation into Polymeric Nanocapsules. WIREs Nanomed. Nanobiotechnol..

[5] Friedrich R.B., Fontana M.C., Beck R.C.R., Pohlmann A.R., Guterres S.S. (2008). Development and Physicochemical Characterization of Dexamethasone-Loaded Polymeric Nanocapsule Suspensions. Quim. Nova.

[6] Siddique M.I., Katas H., Amin M.C.I.M., Ng S.-F., Zulfakar M.H., Buang F., Jamil A. (2015). Minimization of Local and Systemic Adverse Effects of Topical Glucocorticoids by Nanoencapsulation: In Vivo Safety of Hydrocortisone–Hydroxytyrosol Loaded Chitosan Nanoparticles. J. Pharm. Sci..

[7] Md S., Kuldeep Singh J.K.A., Waqas M., Pandey M., Choudhury H., Habib H., Hussain F., Hussain Z. (2019). Nanoencapsulation of Betamethasone Valerate Using High Pressure Homogenization–Solvent Evaporation Technique: Optimization of Formulation and Process Parameters for Efficient Dermal Targeting. Drug Dev. Ind. Pharm..

[8] Ramos Campos E.V., Proença P.L.D.F., Doretto-Silva L., Andrade-Oliveira V., Fraceto L.F., de Araujo D.R. (2020). Trends in Nanoformulations for Atopic Dermatitis Treatment. Expert. Opin. Drug Deliv..

[9] Sutradhar K.B., Amin M.L. (2013). Nanoemulsions: Increasing Possibilities in Drug Delivery. Eur. J. Nanomed..

[10] Deng S., Gigliobianco M.R., Censi R., Di Martino P. (2020). Polymeric Nanocapsules as Nanotechnological Alternative for Drug Delivery System: Current Status, Challenges and Opportunities. Nanomaterials.

[11] Altammar K.A. (2023). A Review on Nanoparticles: Characteristics, Synthesis, Applications, and Challenges. Front. Microbiol..

[12] Yusuf A., Almotairy A.R.Z., Henidi H., Alshehri O.Y., Aldughaim M.S. (2023). Nanoparticles as Drug Delivery Systems: A Review of the Implication of Nanoparticles’ Physicochemical Properties on Responses in Biological Systems. Polymers.

[13] Wang H., Zhou Y., Sun Q., Zhou C., Hu S., Lenahan C., Xu W., Deng Y., Li G., Tao S. (2021). Update on Nanoparticle-Based Drug Delivery System for Anti-Inflammatory Treatment. Front. Bioeng. Biotechnol..

[14] Rigon C., Marchiori M.C.L., da Silva Jardim F., Pegoraro N.S., Chaves P.d.S., Velho M.C., Beck R.C.R., Ourique A.F., Sari M.H.M., de Oliveira S.M. (2019). Hydrogel Containing Silibinin Nanocapsules Presents Effective Anti-Inflammatory Action in a Model of Irritant Contact Dermatitis in Mice. Eur. J. Pharm. Sci..

[15] Giuliani L.M., Osmari B.F., Camponogara C., Pegoraro N.S., Rechia G.C., Sari M.H.M., Oliveira S.M., Cruz L. (2023). Locust Bean Gum Hydrogel Containing Indole-3-Carbinol Nanocapsules Has Prolonged Cutaneous Anti-Inflammatory Action. J. Drug Deliv. Sci. Technol..

[16] Tan Q., He L., Meng X., Wang W., Pan H., Yin W., Zhu T., Huang X., Shan H. (2021). Macrophage Biomimetic Nanocarriers for Anti-Inflammation and Targeted Antiviral Treatment in COVID-19. J. Nanobiotechnol..

[17] Jebbawi R., Fruchon S., Turrin C.-O., Blanzat M., Poupot R. (2020). Supramolecular and Macromolecular Matrix Nanocarriers for Drug Delivery in Inflammation-Associated Skin Diseases. Pharmaceutics.

[18] Mao L., Wu W., Wang M., Guo J., Li H., Zhang S., Xu J., Zou J. (2021). Targeted Treatment for Osteoarthritis: Drugs and Delivery System. Drug Deliv..

[19] Zhang Z., Yu Y., Zhu G., Zeng L., Xu S., Cheng H., Ouyang Z., Chen J., Pathak J.L., Wu L. (2022). The Emerging Role of Plant-Derived Exosomes-Like Nanoparticles in Immune Regulation and Periodontitis Treatment. Front. Immunol..

[20] Luo W., Bai L., Zhang J., Li Z., Liu Y., Tang X., Xia P., Xu M., Shi A., Liu X. (2023). Polysaccharides-Based Nanocarriers Enhance the Anti-Inflammatory Effect of Curcumin. Carbohydr. Polym..

[21] Pegoraro N.S., Barbieri A.V., Camponogara C., Mattiazzi J., Brum E.S., Marchiori M.C.L., Oliveira S.M., Cruz L. (2017). Nanoencapsulation of Coenzyme Q10 and Vitamin E Acetate Protects against UVB Radiation-Induced Skin Injury in Mice. Colloids Surf. B Biointerfaces.

[22] Camponogara C., Brum E.S., Pegoraro N.S., Brusco I., Brucker N., Oliveira S.M. (2021). Diosmetin, a Novel Transient Receptor Potential Vanilloid 1 Antagonist, Alleviates the UVB Radiation-Induced Skin Inflammation in Mice. Inflammopharmacology.

[23] Yu C.-H., Suh B., Shin I., Kim E.-H., Kim D., Shin Y.-J., Chang S.-Y., Baek S.-H., Kim H., Bae O.-N. (2019). Inhibitory Effects of a Novel Chrysin-Derivative, CPD 6, on Acute and Chronic Skin Inflammation. Int. J. Mol. Sci..

[24] Wittmann M., McGonagle D., Werfel T. (2014). Cytokines as Therapeutic Targets in Skin Inflammation. Cytokine Growth Factor. Rev..

[25] Yao Y., Ravn Jørgensen A.-H., Thomsen S.F. (2020). Biologics for Chronic Inflammatory Skin Diseases: An Update for the Clinician. J. Dermatol. Treat..

[26] Moayedi A., Rezaei K., Moini S., Keshavarz B. (2011). Chemical Compositions of Oils from Several Wild Almond Species. J. Am. Oil Chem. Soc..

[27] Garavaglia J., Markoski M.M., Oliveira A., Marcadenti A. (2016). Grape Seed Oil Compounds: Biological and Chemical Actions for Health. Nutr. Metab. Insights.

[28] Akkaya M.R. (2018). Prediction of Fatty Acid Composition of Sunflower Seeds by Near-Infrared Reflectance Spectroscopy. J. Food Sci. Technol..

[29] El Riachy M., Hamade A., Ayoub R., Dandachi F., Chalak L. (2019). Oil Content, Fatty Acid and Phenolic Profiles of Some Olive Varieties Growing in Lebanon. Front. Nutr..

[30] Pegoraro N.S., Camponogara C., Gehrcke M., Giuliani L.M., da Silva D.T., Maurer L.H., Dias P., Emanuelli T., Cruz L., Oliveira S.M. (2020). Oleic Acid-Containing Semisolid Dosage Forms Exhibit In Vivo Anti-Inflammatory Effect via Glucocorticoid Receptor in a UVB Radiation-Induced Skin Inflammation Model. Inflammopharmacology.

[31] Pegoraro N.S., Camponogara C., Cruz L., Oliveira S.M. (2021). Oleic Acid Exhibits an Expressive Anti-Inflammatory Effect in Croton Oil-Induced Irritant Contact Dermatitis without the Occurrence of Toxicological Effects in Mice. J. Ethnopharmacol..

[32] Fessi H., Puisieux F., Devissaguet J.P., Ammoury N., Benita S. (1989). Nanocapsule Formation by Interfacial Polymer Deposition Following Solvent Displacement. Int. J. Pharm..

[33] Urban M.C.C., Mainardes R.M., Gremião M.P.D. (2009). Development and Validation of HPLC Method for Analysis of Dexamethasone Acetate in Microemulsions. Braz. J. Pharm. Sci..

[34] Borghetti G.S., Knorst M.T. (2006). Desenvolvimento e Avaliação Da Estabilidade Física de Loções O/A Contendo Filtros Solares. Rev. Bras. Ciências Farm..

[35] Rigo L.A., Weber J., de Silva C.B., Beck R.C.R. (2012). Evaluation of the Spreadability of Pharmaceutical or Cosmetic Semisolid Formulations Using Scanned Images. Lat. Am. J. Pharm..

[36] Osmari B.F., Giuliani L.M., Reolon J.B., Rigo G.V., Tasca T., Cruz L. (2020). Gellan Gum-Based Hydrogel Containing Nanocapsules for Vaginal Indole-3-Carbinol Delivery in Trichomoniasis Treatment. Eur. J. Pharm. Sci..

[37] Hobson D.W., Hobson D.W. (1991). Dermal and Ocular Toxicology: Fundamentals and Methods.

[38] Laneri S., Sacchi A., Fratter A., Bertin W., Bino A., Vertuani S., Manfredini S. (2017). Self-Nanoemulsifying System in the Accumulation of Resveratrol and *N-* Acetylcysteine in the Epidermis and Dermis. Nat. Prod. Commun..

[39] McGrath J.C., Lilley E. (2015). Implementing guidelines on reporting research using animals (ARRIVE etc.): New requirements for publication in BJP. Br. J. Pharmacol..

[40] University of Iowa (2020). Vertebrate Animal Research—Anesthesia Guideline.

[41] Oliveira S.M., Silva C.R., Wentz A.P., Paim G.R., Correa M.S., Bonacorso H.G., Prudente A.S., Otuki M.F., Ferreira J. (2014). Antinociceptive Effect of 3-(4-Fluorophenyl)-5-Trifluoromethyl-1H-1-Tosylpyrazole. A Celecoxib Structural Analog in Models of Pathological Pain. Pharmacol. Biochem. Behav..

[42] Camponogara C., Silva C.R., Brusco I., Piana M., Faccin H., de Carvalho L.M., Schuch A., Trevisan G., Oliveira S.M. (2019). Nasturtium Officinale R. Br. Effectively Reduces the Skin Inflammation Induced by Croton Oil via Glucocorticoid Receptor-Dependent and NF-ΚB Pathways without Causing Toxicological Effects in Mice. J. Ethnopharmacol..

[43] Vaughn A.R., Clark A.K., Sivamani R.K., Shi V.Y. (2018). Natural Oils for Skin-Barrier Repair: Ancient Compounds Now Backed by Modern Science. Am. J. Clin. Dermatol..

[44] Flores M., Saravia C., Vergara C., Avila F., Valdés H., Ortiz-Viedma J. (2019). Avocado Oil: Characteristics, Properties, and Applications. Molecules.

[45] Sales-Campos H., Reis de Souza P., Crema Peghini B., Santana da Silva J., Ribeiro Cardoso C. (2013). An Overview of the Modulatory Effects of Oleic Acid in Health and Disease. Mini Rev. Med. Chem..

[46] Chen C.-Y., Lee Y.-H., Chang S.-H., Tsai Y.-F., Fang J.-Y., Hwang T.-L. (2019). Oleic Acid-Loaded Nanostructured Lipid Carrier Inhibits Neutrophil Activities in the Presence of Albumin and Alleviates Skin Inflammation. Int. J. Nanomed..

[47] Lio P.A., Lee M., LeBovidge J., Timmons K.G., Schneider L. (2014). Clinical Management of Atopic Dermatitis: Practical Highlights and Updates from the Atopic Dermatitis Practice Parameter 2012. J. Allergy Clin. Immunol. Pract..

[48] Sabat R., Wolk K., Loyal L., Döcke W.-D., Ghoreschi K. (2019). T Cell Pathology in Skin Inflammation. Semin. Immunopathol..

[49] Patra J.K., Das G., Fraceto L.F., Campos E.V.R., del Rodriguez-Torres M.P., Acosta-Torres L.S., Diaz-Torres L.A., Grillo R., Swamy M.K., Sharma S. (2018). Nano Based Drug Delivery Systems: Recent Developments and Future Prospects. J. Nanobiotechnol..

[50] Lee J.H., Yeo Y. (2015). Controlled Drug Release from Pharmaceutical Nanocarriers. Chem. Eng. Sci..

[51] Baryakova T.H., Pogostin B.H., Langer R., McHugh K.J. (2023). Overcoming Barriers to Patient Adherence: The Case for Developing Innovative Drug Delivery Systems. Nat. Rev. Drug Discov..

[52] Maqbool M.A., Mishra M.K., Pathak S., Kesharwani A., Kesharwani A. (2017). Semi Solid Dosage Forms Manufacturing: Tools, Critical Process Parameters, Strategies, Optimization and Recent Advances. Indo Am. J. Pharm. Res..

[53] Pahri R. (2020). Recent Advances in the Development of Semisolid Dosage Forms. Pharmaceutical Drug Product Development and Process Optimization.

[54] Garg A., Aggarwal D., Garg S., Singla A.K. (2002). Spreading of Semisolid Formulations: An Update. Pharm. Technol..

[55] Marto J., Ruivo E., Lucas S.D., Gonçalves L.M., Simões S., Gouveia L.F., Felix R., Moreira R., Ribeiro H.M., Almeida A.J. (2018). Starch Nanocapsules Containing a Novel Neutrophil Elastase Inhibitor with Improved Pharmaceutical Performance. Eur. J. Pharm. Biopharm..

[56] Kaur A., Katiyar S.S., Kushwah V., Jain S. (2017). Nanoemulsion Loaded Gel for Topical Co-Delivery of Clobitasol Propionate and Calcipotriol in Psoriasis. Nanomedicine.

[57] de Lima C.S.A., Varca J.P.R.O., Alves V.M., Nogueira K.M., Cruz C.P.C., Rial-Hermida M.I., Kadłubowski S.S., Varca G.H.C., Lugão A.B. (2022). Mucoadhesive Polymers and Their Applications in Drug Delivery Systems for the Treatment of Bladder Cancer. Gels.

[58] Hamdi N.A.M., Sabari N.H.M., Ismail A.F.H., Azmi N.A. (2023). An Insight into the Use and Advantages of Carbopol in Topical Mucoadhesive Drug Delivery System: A Systematic Review. J. Pharm..

[59] Carvalho F.C., Bruschi M.L., Evangelista R.C., Gremião M.P.D. (2010). Mucoadhesive Drug Delivery Systems. Braz. J. Pharm. Sci..

[60] Kumar A., Naik P.K., Pradhan D., Ghosh G., Rath G. (2020). Mucoadhesive Formulations: Innovations, Merits, Drawbacks, and Future Outlook. Pharm. Dev. Technol..

[61] Saghazadeh S., Rinoldi C., Schot M., Kashaf S.S., Sharifi F., Jalilian E., Nuutila K., Giatsidis G., Mostafalu P., Derakhshandeh H. (2018). Drug Delivery Systems and Materials for Wound Healing Applications. Adv. Drug Deliv. Rev..

[62] Jacob S., Nair A.B., Shah J., Sreeharsha N., Gupta S., Shinu P. (2021). Emerging Role of Hydrogels in Drug Delivery Systems, Tissue Engineering and Wound Management. Pharmaceutics.

[63] Huang Y., Leobandung W., Foss A., Peppas N.A. (2000). Molecular Aspects of Muco- and Bioadhesion. J. Control. Release.

[64] Guerra K.C., Crane J.S. (2023). Sunburn 2023. StatPearls.

[65] Dhar S., Seth J., Parikh D. (2014). Systemic Side-Effects of Topical Corticosteroids. Indian J. Dermatol..

[66] Lan C.-C.E., Wu C.-S., Huang S.-M., Wu C.-H., Lai H.-C., Peng Y.-T., Hou P.-S., Yang H.-J., Chen G.-S. (2016). Irradiance-Dependent UVB Photocarcinogenesis. Sci. Rep..

[67] Peng Z., Chen B., Zheng Q., Zhu G., Cao W., Qin X., Zhang C. (2020). Ameliorative Effects of Peptides from the Oyster (Crassostrea Hongkongensis) Protein Hydrolysates against UVB-Induced Skin Photodamage in Mice. Mar. Drugs.

[68] Kalyan Kumar G., Dhamotharan R., Kulkarni N.M., Mahat M.Y.A., Gunasekaran J., Ashfaque M. (2011). Embelin Reduces Cutaneous TNF-α Level and Ameliorates Skin Edema in Acute and Chronic Model of Skin Inflammation in Mice. Eur. J. Pharmacol..

[69] Cho B.O., Che D.N., Shin J.Y., Kang H.J., Kim J.H., Kim H.Y., Cho W.G., Jang S. (2017). Il Ameliorative Effects of Diospyros Lotus Leaf Extract against UVB-Induced Skin Damage in BALB/c Mice. Biomed. Pharmacother..

[70] Németh T., Mócsai A. (2012). The Role of Neutrophils in Autoimmune Diseases. Immunol. Lett..

[71] Lu Y., Zhang Z., Xiong X., Wang X., Li J., Shi G., Yang J., Zhang X., Zhang H., Hong J. (2012). Glucocorticoids Promote Hepatic Cholestasis in Mice by Inhibiting the Transcriptional Activity of the Farnesoid X Receptor. Gastroenterology.

[72] Lisakovska O., Shymanskyy I., Mazanova A., Khomenko A., Veliky M. (2017). Vitamin D_3_ Protects against Prednisolone-Induced Liver Injury Associated with the Impairment of the Hepatic NF-ΚB/INOS/NO Pathway. Biochem. Cell Biol..

